# Histone H2A Nuclear/Cytoplasmic Trafficking Is Essential for Negative Regulation of Antiviral Immune Response and Lysosomal Degradation of TBK1 and IRF3

**DOI:** 10.3389/fimmu.2021.771277

**Published:** 2021-11-18

**Authors:** Xiao Man Wu, Hong Fang, Jie Zhang, Yong Hong Bi, Ming Xian Chang

**Affiliations:** ^1^ State Key Laboratory of Freshwater Ecology and Biotechnology, Institute of Hydrobiology, Chinese Academy of Sciences, Wuhan, China; ^2^ College of Advanced Agricultural Sciences, University of Chinese Academy of Sciences, Beijing, China; ^3^ Innovation Academy for Seed Design, Chinese Academy of Sciences, Wuhan, China

**Keywords:** histone H2A, nuclear/cytoplasmic trafficking, negative regulation, RLR signaling, SVCV infection

## Abstract

Histone H2A is a nuclear molecule tightly associated in the form of the nucleosome. Our previous studies have demonstrated the antibacterial property of piscine H2A variants against gram-negative bacteria *Edwardsiella piscicida* and Gram-positive bacteria *Streptococcus agalactiae.* In this study, we show the function and mechanism of piscine H2A in the negative regulation of RLR signaling pathway and host innate immune response against spring viremia of carp virus (SVCV) infection. SVCV infection significantly inhibits the expression of histone H2A during an early stage of infection, but induces the expression of histone H2A during the late stage of infection such as at 48 and 72 hpi. Under normal physiological conditions, histone H2A is nuclear-localized. However, SVCV infection promotes the migration of histone H2A from the nucleus to the cytoplasm. The *in vivo* studies revealed that histone H2A overexpression led to the increased expression of SVCV gene and decreased survival rate. The overexpression of histone H2A also significantly impaired the expression levels of those genes involved in RLR antiviral signaling pathway. Furthermore, histone H2A targeted TBK1 and IRF3 to promote their protein degradation *via* the lysosomal pathway and impair the formation of TBK1-IRF3 functional complex. Importantly, histone H2A completely abolished TBK1-mediated antiviral activity and enormously impaired the protein expression of IRF3, especially nuclear IRF3. Further analysis demonstrated that the inhibition of histone H2A nuclear/cytoplasmic trafficking could relieve the protein degradation of TBK1 and IRF3, and blocked the negative regulation of histone H2A on the SVCV infection. Collectively, our results suggest that histone H2A nuclear/cytoplasmic trafficking is essential for negative regulation of RLR signaling pathway and antiviral immune response in response to SVCV infection.

## Highlights

1. SVCV infection promotes histone H2A nuclear/cytoplasmic trafficking.
2. Histone H2A facilitates SVCV replication.
3. Histone H2A interacts with TBK1 and IRF3 to impair the formation of TBK1-IRF3 complex.
4. Histone H2A promotes the protein degradations of TBK1 and IRF3 *via* the lysosomal pathway.

## Introduction

Pathogen-associated molecular patterns (PAMPs) are recognized by evolutionarily conserved host sensors known as pattern recognition receptors (PRRs), which include peptidoglycan recognition proteins (PGRPs), Toll-like receptors (TLRs), RIG-I-like receptors (RLRs), NOD-like receptors (NLRs), C-type lectin receptors (CLRs) and DNA receptors (1–3). Among them, RLRs consisting of RIG-I (retinoic acid-inducible gene I), MDA5 (melanoma differentiation associated factor 5) and LGP2 (laboratory of genetics and physiology 2) are key sensors for virus infection, which mediate the transcriptional induction of type I interferons (IFNs) and interferon-stimulated genes (ISGs) to establish an antiviral immune response (4). It is now well known that the activated RLR signaling by interacting with the adapter protein MAVS leads to initiation of a signaling cascade that includes activation of TBK1 and IKKϵ protein kinases, which ultimately phosphorylate and activate IRF3 and NF-κB transcription factors. Following this activation, IRF3 and NF-κB translocate from the cytoplasm to the nucleus and induce transcription of IFNs and ISGs (5). RLR signaling is found conservatively present in teleost fish and mammals (6, 7).

Besides PAMPs, PRRs are also activated by damage-associated molecular patterns (DAMPs), which are host nuclear or cytoplasmic non-pathogen molecules and utilize the same signal transduction pathways to activate the immune system (8). DAMPs, sometimes termed alarmins or danger signals, are released from cells in response to exogenous and endogenous stressors such as injury or cellular death. DAMPs can also be secreted following PAMP stimulation (9). Protein sources of DAMPs include high mobility group box 1 (HMGB1), heat shock proteins (HSPs), extracellular cold-inducible RNA-binding protein (eCIRP), S100, serum amyloid A (SAA) and histones, whereas non-protein sources of DAMPs include ATP, uric acid, heparin sulfate, genomic and mitochondrial DNA, and extracellular RNAs (9, 10). Among them, histones are located mainly in the nucleus, and have been suggested as important DAMPs by binding to PRRs once they are released to the extracellular space or translocate to the cytoplasm following infection, sterile inflammation and various types of cell death (9–12). In response to stress such as hypoxia, chemical agents and infection, histones H1, H2A, H2B, H3 and H4 are frequently detected at the cell surface or cytoplasm. Extracellular histones could bind to TLRs receptors and induce NALP3 inflammation activation (13).

In teleost fish, the antibacterial properties of piscine H2A variants against gram-negative bacteria *Edwardsiella piscicida* and Gram-positive bacteria *Streptococcus agalactiae* have been investigated in zebrafish and/or grass carp (14–16). Significantly, *S. agalactiae* infection could induce the release of histone H2A. In the case of *S. agalactiae* infection, histone H2A was colocalized with NLR NOD1 both in the cytoplasm and cell nucleus. Histone H2A variant zfH2A-6 also could interact with NOD1 and cooperate with NOD1 to inhibit the proliferation of *S. agalactiae* (16). However, the function of piscine H2A in viral infection is still unclear.

In the present study, we firstly describe the functional characterization of piscine H2A in response to viral infection. Similar to *S. agalactiae* infection, SVCV infection induced the migration of histone H2A from the nucleus to the cytoplasm. Different from the antibacterial roles of histone H2A in bacterial infection, histone H2A negatively regulated antiviral immune response against SVCV infection. Functionally, the cytoplasmic histone H2A degraded TBK1 and IRF3 *via* the lysosomal pathway to impair the formation of TBK1-IRF3 functional complex and the expression level of nuclear IRF3 protein. All these findings shed light on a novel strategy that histone H2A is utilized by SVCV to inhibit host immune response.

## Materials and Methods

### Ethics Statement

All animal experiments were conducted in accordance with the Guiding Principles for the Care and Use of Laboratory Animals and were approved by the Institute of Hydrobiology, Chinese Academy of Sciences (Approval ID: IHB 2013724).

### Cell, Virus, and Reagents

Epithelioma papulosum cyprini (EPC) and zebrafish embryonic fibroblast (ZF4) cells were maintained at 28°C in 5% CO_2_ in medium 199 (Hyclone) and DMEM/F12 (1:1) medium (Invitrogen) supplemented with 10% FBS, 100 U/mL penicillin and 100 mg/mL streptomycin, respectively. Spring viremia of carp virus (SVCV) was propagated in EPC cells. MG132 (S2619) and 3-MA (S2767) were purchased from Selleck. Chloroquine (C6628) and poly I:C (P9582) were purchased from Sigma-Aldrich. Leptomycin B (9676S) was purchased from Cell Signaling Technology.

### Plasmids Construction and Transfection

The expression plasmids including pcDNA3.1-MDA5, pcDNA3.1-RIG-I, p3×FLAG-MAVS, p3×FLAG-TBK1, pTurbo-TBK1-GFP, p3×FLAG-IRF3, pTurbo-IRF3-GFP and p3×FLAG-H2A were described previously (14, 17–20). Zebrafish histone H2A-GFP plasmid was generated using the primer pairs H2AF/H2AR, which were the same primer pairs for constructing p3×FLAG-H2A plasmid (14), and inserted into the pTurboGFP-N plasmid (Everogen). These plasmids were transfected into EPC cells by Lipofectamine 2000 (Invitrogen) according to the manufacturer’s protocol. Expression plasmids including p3×FLAG, p3×FLAG-TBK1 and/or p3×FLAG-H2A were transfected into ZF4 cells by using the Amaxa Nucleofector II transfection system (Lonza) under program T20 according to the manufacturer’s instructions.

### Fluorescence Microscopy

To visualize the subcellular localization of zebrafish histone H2A, ZF4 cells were seeded onto coverslips in 24-well plates. After 24 h, the cells were infected with SVCV at a multiplicity of infection (MOI) of 1 or left untreated. At 6, 12, 24 and 48 hours post-infection (hpi), the cells were fixed with 4% formaldehyde. To investigate whether zebrafish histone H2A colocalizes with TBK1 and IRF3, EPC cells seeded in 24-well plates were transfected with 250 ng H2A-FLAG and 250 ng TBK1-GFP or IRF3-GFP. After 24 h post-transfection, the cells were infected with SVCV at the MOI of 1. At 24 hpi, the cells were fixed with 4% formaldehyde. For fluorescence microscopy analysis, the cells were permeabilized with 0.2% Triton X-100 for 15 min, and then blocked with 5% BSA for 1 h. Next, the cells were incubated in the primary antibody anti-H2A (1:200; 12349S, CST) overnight at 4°C, the secondary antibody goat anti-rabbit IgG conjugated to fluorophore Alexa Fluor 488 (1:500; A-11034, Invitrogen) for 1 h at room temperature, and finally stained with DAPI (1 μg/mL) for 15 min. The coverslips were observed with a confocal microscope (SP8; Lecia).

### Preparation of Nuclear and Cytoplasmic Fractions

To investigate the effects of different multiplicity of infection on zebrafish histone H2A subcellular localization, 1×10^6^ ZF4 cells were cultured in 6-well plates overnight and then infected with SVCV at an MOI of 0.1, 1, 5 and 10. At 24 hpi, the cells were harvested and used for preparation of nuclear and cytoplasmic extracts using a Subcellular Protein Fractionation Kit (Thermo Fisher Scientific).

To investigate the effects of different stressors on zebrafish histone H2A subcellular localization, 1×10^6^ ZF4 cells or EPC cells passed in 6-well plates overnight were infected with SVCV at an MOI of 1 or stimulated with poly I:C (10 μg/mL) or left untreated. At 6, 24 and 48 hpi, the cells were harvested and used for preparation of nuclear and cytoplasmic extracts.

To investigate the effects of histone H2A on the protein degradations of cytoplasmic and/or nuclear IRF3, 1×10^6^ EPC cells seeded in 6-well plates overnight were transfected with various plasmids with the indicated combination. After 24 h post-transfection, the cells were infected with SVCV at an MOI of 1. At 24 hpi, the cells were harvested and used for preparation of nuclear and cytoplasmic extracts.

To investigate the effects of inhibition of histone H2A nuclear/cytoplasmic trafficking on the protein degradations of TBK1 and IRF3, 1×10^6^ EPC cells seeded in 6-well plates overnight were transfected with TBK1-FLAG or IRF3-FLAG (1500 ng/well) and H2A-GFP (800 ng/well). After 36 h post-transfection, the cells were treated with LMB at the concentration of 60 nm, 80 nm, 100 nm or left untreated. After 12 h, the cells were harvested and used for preparation of nuclear and cytoplasmic extracts.

Purity of the fractions was determined by Western blotting using polyclonal anti-tubulin (1:5000; ab6046; Abcam) and anti-HDAC1 (1:5000; ab41407; Abcam) antibodies (Abs). The protein expression of histone H2A was examined using monoclonal anti-H2A (1:1000; 12349S, CST) Ab.

### Luciferase Activity Assay

To examine the effect of histone H2A on the promoter activities of IFNs after poly I:C stimulation, EPC cells seeded overnight in 24-well plates at 3×10^5^ cells per well were cotransfected with the FLAG and/or p3×FLAG-H2A with the indicated concentration, 250 ng DrIFN1pro-luc or DrIFN3pro-luc reporter plasmid and 25 ng Renilla luciferase internal control reporter vector (Promega). After 24 h transfection, the cells were stimulation with poly I:C at a final concentration of 2 μg/mL or left untreated. Another 24 h later, the cells were lysed and used for luciferase activity assay by Dual-Luciferase Reporter Assay System (Promega). To examine the effect of histone H2A on the promoter activities of IFNs mediated by the normal form of RIG-I, MDA5, MAVS, TBK1 and IRF3, EPC cells seeded overnight were transfected with 200 ng various plasmids with the indicated combination. At 48 h post-transfection, the cells were washed with PBS, lysed with 100 μL of lysis buffer and measured luciferase activity. All the experiments were performed in triplicate.

### SVCV Infection in Zebrafish Larvae

In order to investigate the inducible expression of zebrafish histone H2A in response to SVCV infection, zebrafish larvae at 4 dpf (days post-fertilization) were exposed to 2×10^6^ PFU/ml SVCV in a total volume of 5 mL water according to the methods previously reported (21) or left untreated. After immersion in the SVCV suspension for 24 h or left untreated, zebrafish larvae were maintained in 70 mm sterile disposable petri dishes with the supplemented 20 mL water. 30~40 larvae per group collected at 6, 12, 24, 48 and 72 hpi were used for RNA extraction and quantivative RT-PCR (qRT-PCR).

To determine the role of zebrafish histone H2A in SVCV infection, 100 ng/μL p3×FLAG empty plasmid or p3×FLAG-H2A was microinjected into zebrafish embryos at the one-cell stage. The typical injected volume was 2 nL. At 4 dpf, the zebrafish larvae were divided into 2 groups and used for SVCV infection (2×10^6^ PFU/mL) or left untreated. The numbers of surviving larvae were counted daily for 6 dpi. GraphPad Prism 7 was used to generate survival curves, and the log-rank test was used to test differences in survival with WT zebrafish microinjected with p3×FLAG as the control group. To examine the expression of SVCV-N gene and confirm the overexpression of histone H2A in zebrafish larvae, 20 larvae per group were collected at 24 and 48 hpi, and used for RNA extraction and qRT-PCR. To determine the effect of histone H2A overexpression on the transcriptional regulation of antiviral genes involved in RLR signaling, 20~30 zebrafish embryos or larvae microinjected with p3×FLAG or H2A-FLAG were collected at 1, 3 and 7 dpf for RNA extraction and qRT-PCR.

### SVCV Infection in ZF4 and EPC Cells

To determine the regulatory effect of zebrafish histone H2A in TBK1-mediated antiviral activity and TBK1-mediated induction of IFNs and ISGs, 1×10^6^ ZF4 cells were transfected with 2 μg p3×FLAG, TBK1-FLAG and/or H2A-FLAG alone or in combination of any two. At 36 h post-transfection, these cells were used for SVCV infection at an MOI of 1. At 48 hpi, the supernatants of transfected cells were collected for the determination of virus titers by a standard TCID50 method. The cell pellets of transfected cells were collected and used for RNA extraction. The overexpression of histone H2A in ZF4 cells and the expression of SVCV genes, IFNs and ISGs were examined by qRT-PCR.

The transfection efficiency of ZF4 cells was rather low, especially in the case of transfection of multiple plasmids. Therefore, EPC cells with higher transfection efficiency were used for overexpression experiments. Since examining the effect of histone H2A on the protein degradations of TBK1 and IRF3 was performed in EPC cells, we used EPC cells to further investigate the effects of inhibition of histone H2A nuclear/cytoplasmic trafficking on the histone H2A-mediated negative regulation of antiviral immune response in response to SVCV infection. 3×10^5^ EPC cells seeded in 24-well plates were transfected with p3×FLAG or H2A-FLAG for 36 h, then treated with LMB at the concentration of 100 nm for 12 h or left untreated. At 48 h posttransfection, these cells were infected with SVCV at an MOI of 1. Another 24 h later, the supernatants of infected cells were collected for the determination of virus titers by a standard TCID50 method. The cell pellets of infected cells were collected for RNA extraction. The expressions of SVCV genes including SVCV-N, SVCV-P and SVCV-G were examined by qRT-PCR.

### qRT-PCR

Total RNA was extracted from ZF4 cells, EPC cells or zebrafish larvae using TRIzol (Invitrogen). First-strand cDNAs were synthesized using the RevertAid First-Strand cDNA Synthesis Kit (Thermo Scientific). qRT-PCR was performed using Fast SYBR Green PCR Master mix (Bio-Rad) on a CFX96 Touch Real-Time PCR Detection System (Bio-Rad) in 96-well plates according to the procedure as follows: preincubation at 95°C for 5 min, then 45 cycles at 95°C for 15 s, 56°C for 20 s, and 72°C for 20 s. Each sample was tested in triplicate. All primers are described by previous studies (14, 22–25). The relative mRNA expression was calculated by normalizing the Ct values of target genes against the reference housekeeping gene GAPDH. The data were analyzed using the 2^-△△Ct^ method.

### Co-Immunoprecipitation (Co-IP) and Western Blotting (WB)

To determine whether histone H2A interacted with TBK1 and IRF3 to impair the formation of TBK1-IRF3, EPC cells seeded in T25 culture bottles overnight were cotransfected with 5000 ng various plasmid with the indicated combinations. After 48 h post-transfection, the cells were lysed in IP lysis buffer containing Protease Inhibitor Cocktail. The immune complexes were captured using FLAG-tagged Protein Immunoprecipitation Kit according to the manufacturer’s protocol, washed six times with ice-cold washing buffer and examined by Western Blotting using anti-pTurboGFP (1:5000; AB513; Evrogen) and anti-FLAG (1:5000; F3165; Sigma-Aldrich) Abs. Input proteins were analyzed using anti-GAPDH (1:5000; Proteintech), anti-FLAG and anti-pTurboGFP Abs.

To examine the effect of histone H2A on the protein degradations of TBK1 and IRF3, 1×10^6^ EPC cells seeded in 6-well plates overnight were transfected with the indicated plasmids and concentration. To determine the exact mechanism of histone H2A on the protein degradations of TBK1 and IRF3, 1×10^6^ EPC cells seeded in 6-well plates overnight were transfected with 1500 ng TBK1-FLAG or IRF3-FLAG and 800 ng H2A-GFP. After 48 h transfection, the cells were treated with MG132, 3-MA, NH_4_CL and Chloroquine (CQ) at the indicated concentration for another 6 h or left untreated. The cells were lysed in RIPA lysis buffer containing Protease Inhibitor Cocktail and analyzed by Western blotting with anti-GAPDH, anti-FLAG, and anti-pTurboGFP Abs. Western blotting results were quantified using Quantity One software.

### Statistical Analysis

Data of qRT-PCR were expressed as means ± SD of at least three independent experiments. The significance of results was analyzed by a two-tailed Student’s T-test or a one-way ANOVA (*p < 0.05, **p < 0.01).

## Results

### Poly I:C Stimulation and SVCV Infection Promote Histone H2A Nuclear/Cytoplasmic Trafficking

**Figure 1:**
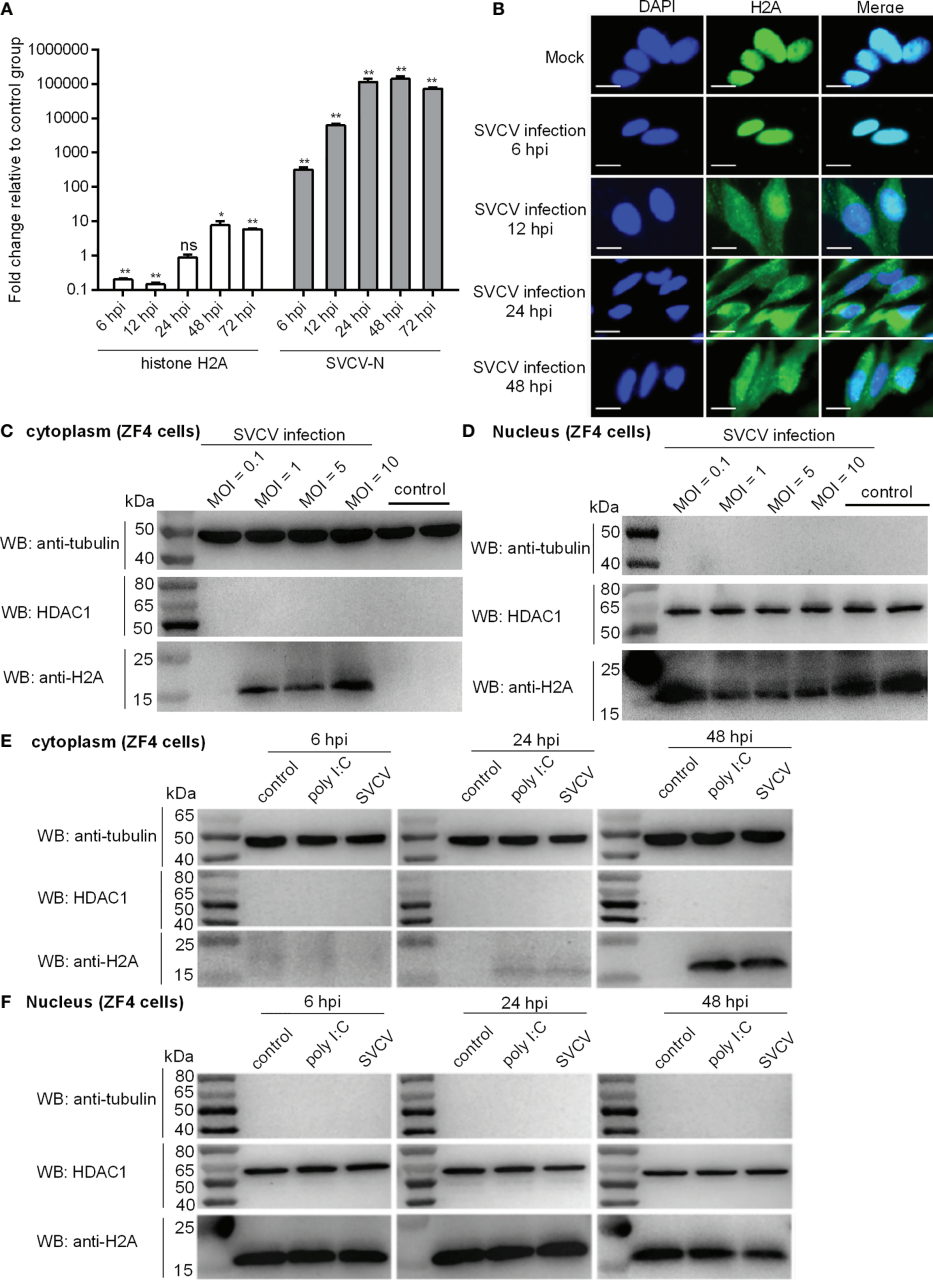
Expression and subcellular localization of zebrafish histone H2A. **(A)** The expressions of histone H2A and SVCV-N in zebrafish larvae in response to SVCV infection. Total RNA was extracted from zebrafish larvae collected at 6, 12, 24, 48 and 72 hpi. Data represented means ± SEM (n = 3), and were tested for statistical significance. *p < 0.05; **p < 0.01; ns, not significant. **(B)** Subcellular localization of zebrafish histone H2A by immunofluorescence analysis in the mock-infected and SVCV-infected ZF4 cells. Scale bar: 10 μm. **(C)** Cytoplasmic fractions from the mock-infected and SVCV-infected ZF4 cells. **(D)** Nuclear fractions from the mock-infected and SVCV-infected ZF4 cells. For **(C, D)**, ZF4 cells cultured in 6-well plates overnight were infected with SVCV at an MOI of 0.1, 1, 5 and 10. At 24 hpi, the cells were harvested and used for preparation of nuclear and cytoplasmic extracts. **(E)** Cytoplasmic fractions from SVCV-infected, poly I:C-stimulated and untreated ZF4 cells. **(F)** Nuclear fractions from SVCV-infected, poly I:C-stimulated and untreated ZF4 cells. For **(E, F)**, ZF4 cells passed in 6-well plates overnight were infected with SVCV at an MOI of 1 or stimulated with poly I:C (10 μg/mL) or left untreated. At 6, 24 and 48 hpi, the cells were harvested and used for preparation of nuclear and cytoplasmic extracts.

The inducible expression of zebrafish histone H2A (GenBank accession number MK800012) in response to bacterial infection has been characterized in our previous study (14). To determine the effect of SVCV infection on the expression regulation of zebrafish histone H2A, the hatched larvae at 4 dpf were infected with 2×10^6^ PFU/ml SVCV. The expression of SVCV-N gene was the highest at 48 hpi. The increased 317.76-fold, 6208.65-fold, 114440.31-fold, 141146.24-fold and 73062.24 for SVCV-N were observed at 6, 12, 24, 48 and 72 hpi, respectively. Compared with the control larvae without infection, the expression of histone H2A in the larvae infected with SVCV was significantly down-regulated at 6 and 12 hpi, no significant change at 24 hpi, and significantly up-regulated at 48 and 72 hpi (**Figure 1A**).

In order to examine the subcellular localization of histone H2A during viral infection, SVCV-infected cells were fixed at different time points post-infection and processed for analysis by confocal microscopy. At 6 hpi for early time point post-infection, histone H2A was distributed only in the nucleus of infected cells. At 12, 24 and 48 hpi, histone H2A was distributed in both the cytoplasm and nucleus of infected cells (**Figure 1B**).

In order to further determine the effect of SVCV infection on the histone H2A nuclear/cytoplasmic trafficking, SVCV-infected cells with the different MOIs were collected and used for subcellular fractionation. The histone H2A nuclear/cytoplasmic trafficking was obvious only at an MOI of 1, 5 or 10, but not observed at an MOI of 0.1 (**Figures 1C, D**).

To further investigate and confirm histone H2A nuclear/cytoplasmic trafficking in response to different stressors, ZF4 cells were stimulated by poly I:C or infected with SVCV, or left untreated. Compared with the untreated control, the protein expression of histone H2A in the cytoplasm was induced by poly I:C stimulation and SVCV infection weakly at 24 hpi and strongly at 48 hpi (**Figure 1E**). Compared with the untreated control, the protein expression of histone H2A in the nucleus of infected cells was decreased by SVCV infection at 48 hpi (**Figure 1F**). Histone H2A nuclear/cytoplasmic trafficking in response to poly I:C stimulation and SVCV infection was also confirmed in EPC cells (**Figure S1** in **Supplementary Material**).

### Histone H2A Negatively Regulates Antiviral Response and RLR Signaling

**Figure 2:**
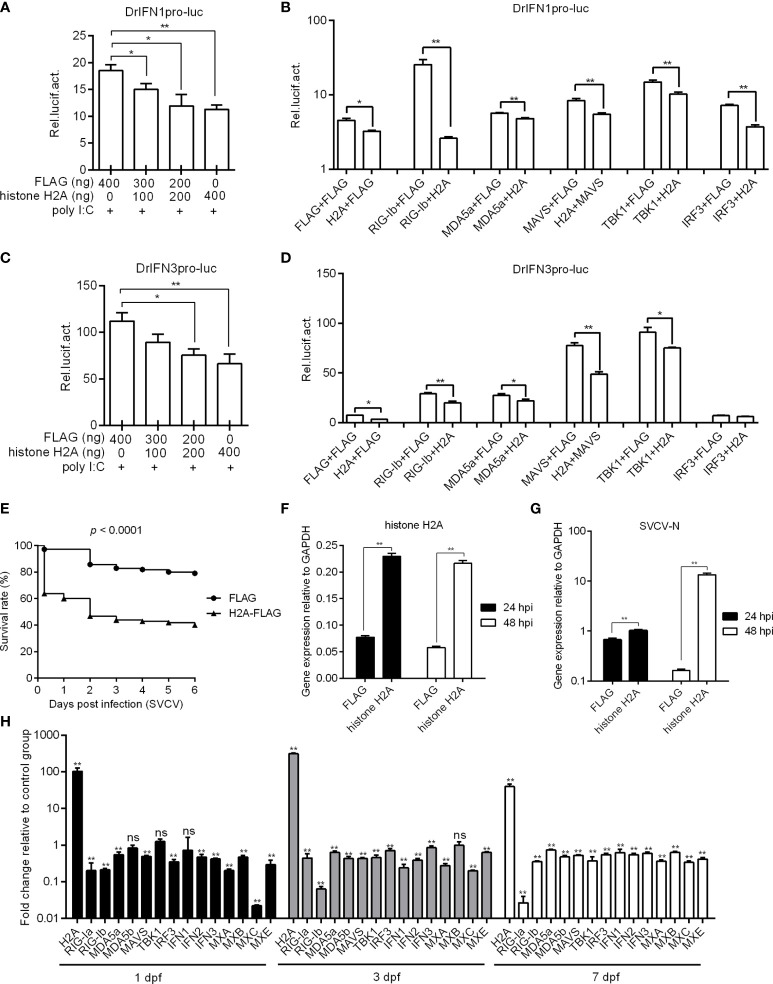
Zebrafish histone H2A negatively regulates RLR signaling. **(A)** Effect of zebrafish histone H2A on the IFN1 promoter activity mediated by poly I:C stimulation. **(B)** Effect of zebrafish histone H2A on the IFN1 promoter activity mediated by RLR signaling pathway. **(C)** Effect of zebrafish histone H2A on the IFN3 promoter activity mediated by poly I:C stimulation. **(D)** Effect of zebrafish histone H2A on the IFN3 promoter activity mediated by RLR signaling pathway. For **(A, C)**, EPC cells transfected with various indicated plasmids and DNA concentration were stimulation with poly I:C. Another 24 h later, the cells were lysed and used for luciferase activity assay. For **(B, D)**, EPC cells were transfected with the indicated combination. At 48 h post-transfection, the cells were used for luciferase activity assay. **(E)** Effect of zebrafish histone H2A on the larvae survival. **(F)** The confirmation of zebrafish histone H2A expression in the histone H2A-overexpressed larvae with SVCV infection. **(G)** Effect of zebrafish histone H2A on the expression of SVCV-N gene. For **(E–G)**, zebrafish larvae microinjected with p3×FLAG or H2A-FLAG were infected with 2×10^6^ PFU/mL SVCV for 24 h. For **(E)**, larvae were monitored for 6 days. For **(F, G)**, larvae were collected at 24 and 48 hpi, and used for qRT-PCR. **(H)** Effect of zebrafish histone H2A on the expression of antiviral genes involved in RLR signaling pathway. Zebrafish larvae microinjected with p3×FLAG or H2A-FLAG were collected at 1, 3 and 7 dpf, and used for qRT-PCR. For **(A–D, F–H)**, data represented means ± SEM (n = 3), and were tested for statistical significance. *p < 0.05; **p < 0.01; ns, not significant.

To investigate the possible role of histone H2A in RLR signaling, the IFN1 and IFN3 promoter activities with or without poly I:C stimulation were firstly investigated in EPC cells by Dual-Luciferase Reporter Assay. The overexpression of histone H2A inhibited the activation of zebrafish IFN1 promoter with the poly I:C stimulation (**Figure 2A**). In the absence of poly I:C stimulation, histone H2A also inhibited the activation of zebrafish IFN1 promoter. The induced activation of zebrafish IFN1 promoter by RIG-I, MDA5, MAVS, TBK1 and IRF3 was significantly inhibited by histone H2A (**Figure 2B**). Similar studies were observed for zebrafish IFN3 promoter, except for no effect of histone H2A on the IRF3-mediated promoter activity (**Figures 2C, D**).

The role of histone H2A overexpression in SVCV infection was further investigated. Compared with zebrafish larvae microinjected with FLAG empty plasmid, SVCV-infected larvae microinjected with histone H2A exhibited reduced survival rate about 40% (**Figure 2E**). The overexpression of histone H2A in zebrafish larvae was confirmed by qRT-PCR (**Figure 2F**), which significantly promoted the expression of *SVCV-N* both at 24 hpi and 48 hpi (**Figure 2G**). The function of histone H2A in the regulation of these genes involved in RLR signaling was investigated by qRT-PCR. The overexpression of histone H2A in zebrafish larvae significantly inhibited the expression of *RIG-Ia*, *RIG-Ib*, *MDA5a*, *MDA5b*, *MAVS*, *IRF3*, *IFN1*, *IFN2*, *IFN3*, *MXA*, *MXB*, *MXC* and *MXE* at 1, 3 and/or 7 dpf (**Figure 2H**). Together these results suggest a negative role for histone H2A in antiviral response and RLR signaling.

### Histone H2A Targets TBK1 and IRF3 to Impair the Formation of TBK1-IRF3 Functional Complex

**Figure 3:**
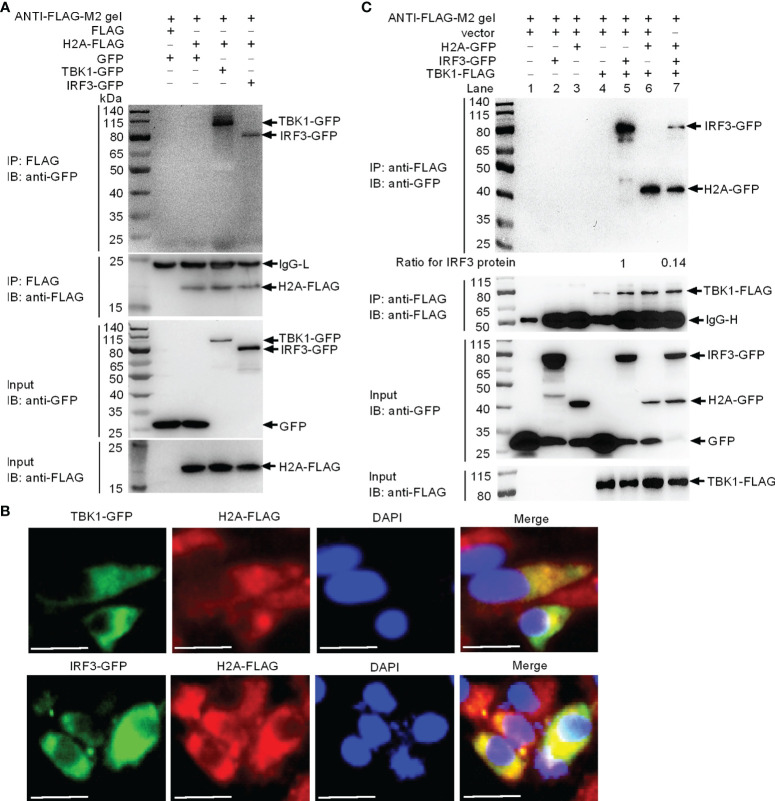
Zebrafish histone H2A interacts with TBK1 and IRF3 to disrupt the formation of TBK1-IRF3 complex. **(A)** Zebrafish histone H2A interacted with TBK1 and IRF3. **(B)** Zebrafish histone H2A colocalized with TBK1 and IRF3. **(C)** Zebrafish histone H2A interacted with TBK1 to inhibit the formation of TBK1-IRF3 complex. For **(A, C)**, co-IP was performed with anti-FLAG-conjugated agarose beads in EPC cells. The cell lysates and bound proteins were analyzed by immunoblotting with the indicated Abs. All experiments were repeated for at least three times with similar results. The expression ratio for IRF3 protein was quantified by Quantity One. For **(B)**, immunofluorescence analysis was performed in EPC cells transfected with H2A-FLAG and TBK1-GFP or IRF3-GFP. Scale bar: 25 μm.

Since the TBK1-IRF3 axis is critical for the antiviral immune response, we investigated if histone H2A was able to interact with TBK1 and IRF3. Immunoprecipitation and immunoblot assays indicated that histone H2A and TBK1 or IRF3 associated together (**Figure 3A**). Immunofluorescence analysis demonstrated that histone H2A colocalized with TBK1 and IRF3 (**Figure 3B**). Next, we investigated whether the interaction between histone H2A and TBK1 or IRF3 might influence the formation of TBK1-IRF3 functional complex. Anti-FLAG conjugated agarose beads were used to precipitate TBK1-FLAG-containing protein complex, and found IRF3-GFP (using anti-GFP antibody for IP product in lanes 5 and 7 in **Figure 3C**) or histone H2A-GFP (using anti-GFP antibody for IP product in lanes 6 and 7 in **Figure 3C**) in this complex. Compared with IRF3-GFP band for the sample without the addition of histone H2A, the addition of histone H2A significantly decreased TBK1-IRF3 interaction (using anti-GFP antibody for IP product in lane 7 in **Figure 3C**). Collectively, these data suggest that histone H2A interacts with TBK1 and IRF3 to impair the formation of TBK1-IRF3 complex when overexpressed in EPC cells.

### Histone H2A Degrades TBK1 and IRF3 *via* the Lysosomal Pathway

**Figure 4:**
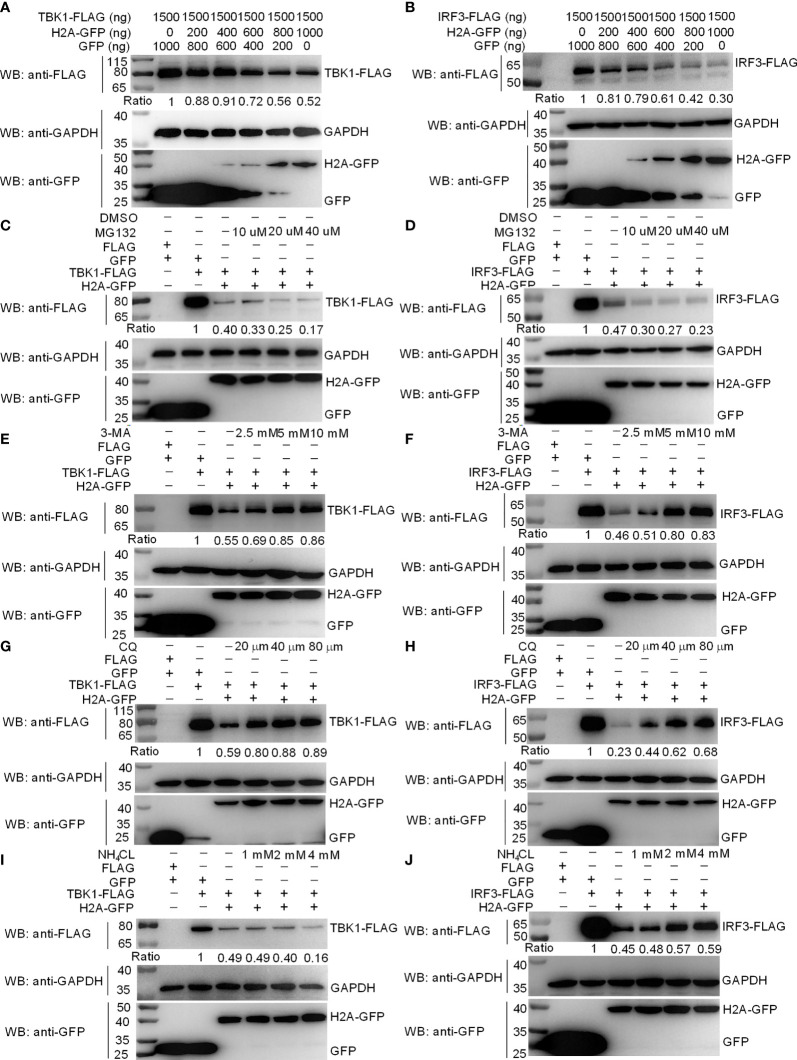
Zebrafish histone H2A degrades TBK1 and IRF3 *via* the lysosomal pathway. **(A)** Effect of zebrafish histone H2A on the protein expression of TBK1. **(B)** Effect of zebrafish histone H2A on the protein expression of IRF3. For **(A, B)**, EPC cells seeded in six-well plates were transfected with various indicated plasmids and DNA concentration. After 48 h post-transfection, cell lysates were analyzed by Western blotting using the indicated Abs. **(C)** Effect of MG132 on the histone H2A-mediated protein degradation of TBK1. **(D)** Effect of MG132 on the histone H2A-mediated protein degradation of IRF3. **(E)** Effect of 3-MA on the histone H2A-mediated protein degradation of TBK1. **(F)** Effect of 3-MA on the histone H2A-mediated protein degradation of IRF3. **(G)** Effect of CQ on the histone H2A-mediated protein degradation of TBK1. **(H)** Effect of CQ on the histone H2A-mediated protein degradation of IRF3. **(I)** Effect of NH_4_Cl on the histone H2A-mediated protein degradation of TBK1. **(J)** Effect of NH_4_Cl on the histone H2A-mediated protein degradation of IRF3. For **(C–J)**, EPC cells seeded in six-well plates were transfected with various indicated plasmids and DNA concentration. After 48 h post-transfection, cells were treated with DMSO, MG132, 3-MA, CQ or NH_4_Cl with indicated concentration for 6 h. Following this, cell lysates were analyzed by Western blotting using the indicated Abs. The expression ratio for TBK1 or IRF3 protein was quantified by Quantity One. All experiments were repeated for at least three times with similar results.

Since histone H2A impaired the formation of TBK1-IRF3 complex, we next examined whether histone H2A might influence the protein levels of TBK1 and IRF3. Western blotting analysis showed that the amount of TBK1 and IRF3 proteins was significantly decreased by the overexpression of histone H2A (**Figures 4A, B**). To further reveal the mechanisms involved in histone H2A degrading with TBK1 and IRF3 expression during the posttranscriptional process, the effects of MG132 (inhibitor for the proteasome), early autophagy inhibitor 3-methyladenine (3-MA) and late autophagy inhibitor chloroquine (CQ) or ammonium chloride (NH_4_Cl) on histone H2A-mediated degradations of TBK1 and IRF3 were investigated in EPC cells. The degradations of TBK1 and IRF3 were not inhibited by MG132 (**Figures 4C, D**). However, the degradations of TBK1 and IRF3 were partly inhibited by 3-MA and CQ, which were dependent of the dose variation (**Figures 4E**–**H**). In addition, NH_4_Cl treatment only partly inhibited histone H2A-mediated degradation of IRF3, but not for TBK1 (**Figures 4I, J**). These data demonstrate that histone H2A degrades TBK1 and IRF3 *via* autophagosome-lysosome pathway.

### Histone H2A Abolishes TBK1-Mediated Antiviral Activity and Inhibits TBK1-Mediated Induction of IFN1 and ISGs

**Figure 5:**
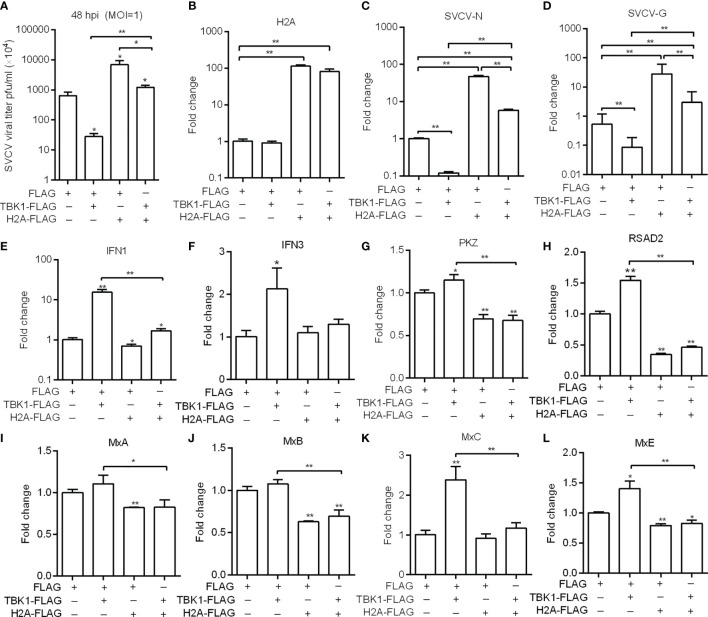
Zebrafish histone H2A inhibits the effects mediated by TBK1. **(A)** Effect of zebrafish histone H2A on the TBK1-mediated antiviral activity. **(B)** The confirmation of zebrafish histone H2A expression in the histone H2A-overexpressed ZF4 cells with SVCV infection. **(C)** Effect of zebrafish histone H2A on the expression of SVCV-N gene mediated by TBK1. **(D)** Effect of zebrafish histone H2A on the expression of SVCV-G gene mediated by TBK1. **(E)** Effect of zebrafish histone H2A on the expression of IFN1 mediated by TBK1. **(F)** Effect of zebrafish histone H2A on the expression of IFN3 mediated by TBK1. **(G)** Effect of zebrafish histone H2A on the expression of PKZ mediated by TBK1. **(H)** Effect of zebrafish histone H2A on the expression of RSAD2 mediated by TBK1. **(I)** Effect of zebrafish histone H2A on the expression of MxA mediated by TBK1. **(J)** Effect of zebrafish histone H2A on the expression of MxB mediated by TBK1. **(K)** Effect of zebrafish histone H2A on the expression of MxC mediated by TBK1. **(L)** Effect of zebrafish histone H2A on the expression of MxE mediated by TBK1. For **(A–L)** ZF4 cells were transfected with the indicated plasmids. At 36 h post-transfection, these cells were used for SVCV infection at an MOI of 1. At 48 hpi, the supernatants of transfected cells were collected for the determination of virus titers. The cell pellets of transfected cells were collected and used for qRT-PCR. Data represented means ± SEM (n = 3), and were tested for statistical significance. *p < 0.05; **p < 0.01. The asterisk above the error bars indicates statistical significance using the group microinjected with empty plasmid as the control group. The asterisk above the bracket indicates statistical significance between the two groups connected by the bracket.

Previous studies have shown that piscine TBK1 plays an important role in the antiviral immune responses against SVCV and GCRV infection (20, 26). We therefore further investigated the role of histone H2A in TBK1-mediated antiviral activity. The supernate of ZF4 cells transfected with TBK1 and/or histone H2A were used for the detection of SVCV viral loads. Compared with the control group transfected with empty plasmids, lower viral loads were observed for ZF4 cells transfected with TBK1, higher viral loads for ZF4 cells co-transfected with histone H2A and TBK1, and the highest viral loads for ZF4 cells transfected with histone H2A (**Figure 5A**). The cell precipitates of ZF4 cells transfected with TBK1 and/or histone H2A were used for the examination of expressions of viral genes. The overexpression of histone H2A was confirmed by qRT-PCR (**Figure 5B**). Compared with the control group transfected with empty plasmids, the expression of SVCV-N or SVCV-G was significantly decreased by TBK1 overexpression, but increased by the coexpression of histone H2A and TBK1 (**Figures 5C, D**). All these data suggest that histone H2A can completely abolish TBK1-mediated antiviral activity.

TBK1 can induce a higher expression of IFNs and ISGs (20, 27). We next investigated whether histone H2A inhibited TBK1-mediated induction of antiviral effector genes in the case of SVCV infection. The overexpression of histone H2A significantly inhibited the expression of *IFN1*, *PKZ*, *RSAD2*, *MxA*, *MxB* and *MxE.* Furthermore, the overexpression of histone H2A significantly inhibited TBK1-mediated induction of IFN1 and ISGs including *PKZ*, *RSAD2*, *MxC* and *MxE* (**Figures 5E**–**L**).

### Histone H2A Impairs the Protein Expression of Cytoplasmic and Nuclear IRF3

**Figure 6:**
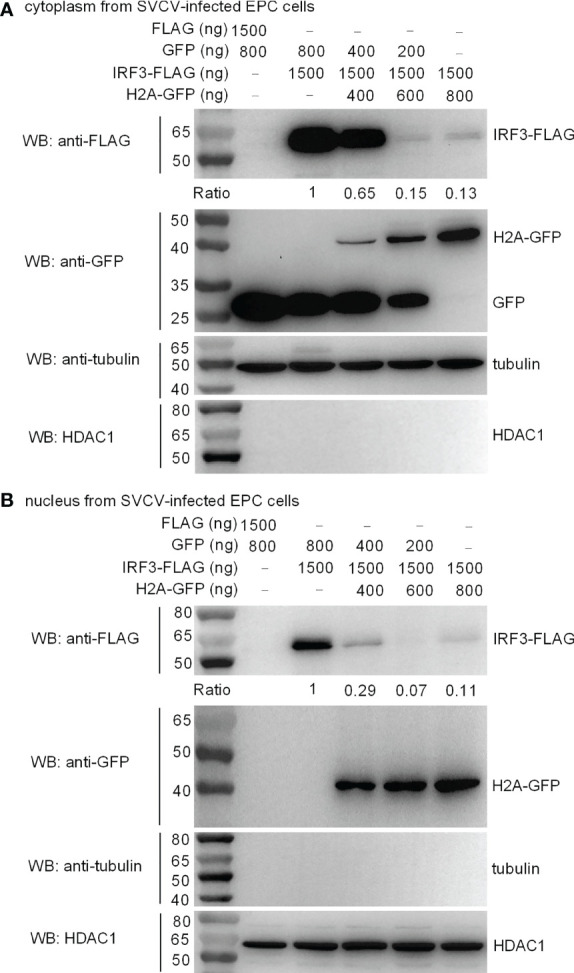
Zebrafish histone H2A impairs the protein expression of cytoplasmic **(A)** and nuclear IRF3 protein **(B)**. For **(A, B)**, EPC cells seeded in 6-well plates were transfected with various indicated plasmids. After 36 h post-transfection, the cells were infected with SVCV. After 12 h, the cells were harvested and used for preparation of nuclear and cytoplasmic extracts. The expression ratio for IRF3 protein was quantified by Quantity One.

IRF3 normally shuttles between nucleus and cytoplasm. To investigate the location of histone H2A-mediated IRF3 degradation, cytoplasmic and nuclear fractions were prepared from SVCV-infected EPC cells transfected with various indicated plasmids. Overexpression of histone H2A was found to degrade cytoplasmic IRF3 in a dose dependent manner (**Figure 6A**). Notably, histone H2A also promoted the degradation of nuclear IRF3 protein (**Figure 6B**).

### Inhibition of Histone H2A Nuclear/Cytoplasmic Trafficking Relieves the Protein Degradations of TBK1 and IRF3, and Blocks the Negative Regulation of Histone H2A in SVCV Infection

**Figure 7:**
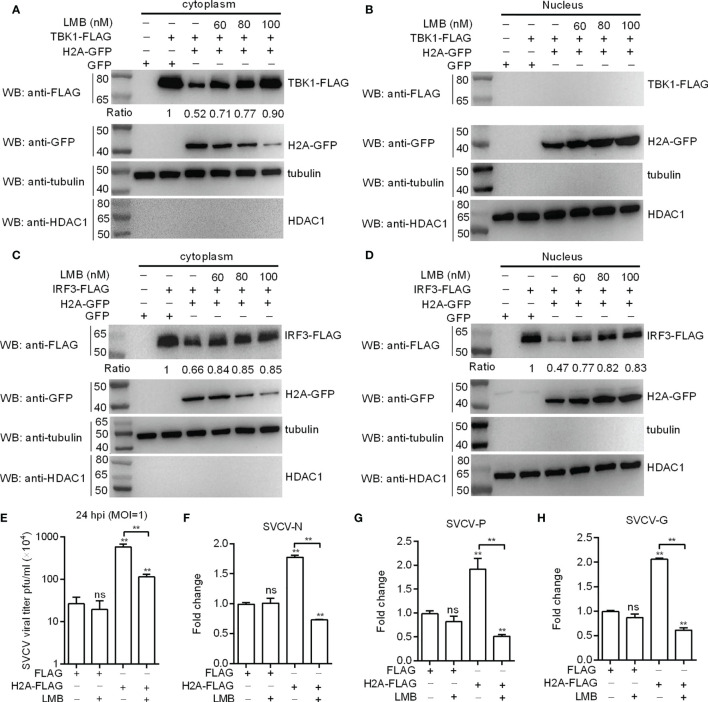
Histone H2A nuclear/cytoplasmic trafficking is essential for the negative regulation of histone H2A. **(A)** Effect of the inhibition of histone H2A nuclear/cytoplasmic trafficking on the protein degradation of cytoplasmic TBK1. **(B)** Effect of the inhibition of histone H2A nuclear/cytoplasmic trafficking on the protein degradation of nuclear TBK1. **(C)** Effect of the inhibition of histone H2A nuclear/cytoplasmic trafficking on the protein degradation of cytoplasmic IRF3. **(D)** Effect of the inhibition of histone H2A nuclear/cytoplasmic trafficking on the protein degradation of nuclear IRF3. For **A–D)**, EPC cells seeded in 6-well plates were transfected with various indicated plasmids. After 36 h post-transfection, the cells were treated with LMB at the indicated concentration or left untreated. After 12 h, the cells were harvested and used for preparation of nuclear and cytoplasmic extracts. The expression ratio for TBK1 or IRF3 protein was quantified by Quantity One. **(E)** Effect of the inhibition of histone H2A nuclear/cytoplasmic trafficking on the histone H2A-mediated SVCV replication. **(F)** Effect of the inhibition of histone H2A nuclear/cytoplasmic trafficking on the expression of SVCV-N mediated by histone H2A. **(G)** Effect of the inhibition of histone H2A nuclear/cytoplasmic trafficking on the expression of SVCV-P mediated by histone H2A. **(H)** Effect of the inhibition of histone H2A nuclear/cytoplasmic trafficking on the expression of SVCV-G mediated by histone H2A. For **(E–H)**, EPC cells seeded in 24-well plates were transfected with p3×FLAG or H2A-FLAG, then treated with LMB or left untreated. After 48 h posttransfection, the cells were infected with SVCV. Another 24 h later, the supernatants of infected cells were collected for the determination of virus titers. The cell pellets of infected cells were collected for qRT-PCR. Data represented means ± SEM (n = 3), and were tested for statistical significance. **p < 0.01; ns, not significant. The asterisk above the error bars indicates statistical significance using the group microinjected with empty plasmid as the control group. The asterisk above the bracket indicates statistical significance between the two groups connected by the bracket.

Histone H2A was translocated into cytoplasm after SVCV infection. To directly confirm the nuclear/cytoplasmic trafficking of histone H2A is required for the protein degradations of TBK1 and IRF3 and the negative regulation of histone H2A in SVCV infection, nuclear export inhibitor LMB was used. LMB treatment (100 nM) largely blocked the nuclear export of histone H2A, and almost completely abolished histone H2A-induced TBK1 degradation in SVCV-infected cells (**Figures 7A, B**). Inhibition of histone H2A nuclear/cytoplasmic trafficking by LMB also inhibited histone H2A-induced IRF3 degradation both in the cytoplasm and the nucleus (**Figures 7C, D**). Furthermore, LMB treatment significantly decreased the SVCV replication mediated by histone H2A in the supernatants of infected cells (**Figure 7E**). Most of all, the expressions of SVCV genes including SVCV-N, SVCV-P and SVCV-G were significantly decreased by the histone H2A and LMB treatment compared with the control group transfected with empty plasmids and the group transfected with histone H2A but without LMB treatment (**Figures 7F**–**H**). Taken together, these results suggest that the cytoplasmic localization of histone H2A is required for the protein degradations of TBK1 and IRF3 and the negative regulation of histone H2A on the SVCV infection.

## Discussion

Histones are localized to the nucleus, and are fundamental structural components of chromatin. However many studies have shown the extrachromosomal functions of histones. Histones are found to translocate to the cytoplasm following infection or in response to various exogenous and endogenous stressors, and mediate functions in the cytoplasm. For example, histone H1.2 showed a time-dependent and X-ray dose-dependent increase in the cytoplasm after X-ray irradiation, and promoted the mitochondrial apoptotic pathway (28). Double-stranded DNA (dsDNA) stimulation induced the aggregation of histone H2B and the interaction between histone H2B and MAVS in the cytoplasm, which triggered antiviral innate immune responses against DNA viruses (29). This study demonstrates for the first time that SVCV, a negative ssRNA virus, induces the translocation of nuclear histone H2A to the cytoplasm. Histone H2A nuclear/cytoplasmic trafficking triggers the protein degradations of TBK1 and IRF3 *via* the lysosomal pathway, which leads to the impaired piscine antiviral response.

It was reported that histones could act as regulators of TLR or NLR signaling. In mice, histone H4 directly interacts with TLR2 and TLR4, and activates TLR2 and TLR4 to induce the production of proinflammatory cytokines in a MyD88-dependent manner (30). Nucleotide binding oligomerzation domain 1 (NOD1), NOD2 and NLRP3 belong to the NLR family. Once released from the nucleosome, extracellular histones have been reported to activate NLRP3 inflammasome (31). Extracellular histone H3, whose stimulation could elevate the expression of NLRP3 and NOD2, caused pyroptosis in sepsis *via* NOD2 and VSIG4/NLRP3 pathways (32). In teleost fish, our previous studies revealed that histone H2A variant cooperated with NOD1 or RIP2 of NLR signaling to induce the transcription of antibacterial PRRs including *NOD1*, *PGRP2*, *PGRP5* and *PGRP6* (14, 16). The present study showed that the overexpression of histone H2A significantly impaired the expression of antiviral RLRs. All these results demonstrate that histones acting as alarmin signals can activate or suppress PRRs such as TLRs, NLRs, RLRs and PGRPs. Furthermore, previous studies revealed that TLR2 and TLR4 may be PRRs for recognizing histones. In mouse peritoneal macrophages (PEMs), the most effective histones for the induction of cytokine and chemokine release were H2A and H2B. However in mouse PEMs exposed to histones, the absence of TLR2 and TLR4 dramatically reduced the cytokine responses (33). Since piscine histone H2A can impair the expression of RLRs, it is interesting to further investigate the effect and mechanisms of histone H2A-mediated ubiquitination in RIG-I antiviral activity.

Histone variants allow for greater control of DNA replication, repair or transcription. The variants of histone H2A include H2A.X, H2A.Z, MacroH2A, H2A.Bbd, etc (34). Among them, H2A.Z is the most studied variant of H2A. H2A.Z has been associated with both transcription activation and transcription inhibition (35). In human cells, H2A.Z acts as a negative regulator of antiviral responses *via* GCN5 and BRD2 (36). In shrimp *Penaeus vannamei*, the white spot syndrome virus (WSSV) decreased the expression of histones H2A and H4 to promote viral replication (37). Although the lysine rich histones H2A and H2B were less potent compared to the arginine rich histones H3, H3.3 and H4, all histones inhibited infectivity of influenza A H3N2 strain Phil82 in mammals (38). Here, we provided evidence that zebrafish histone H2A negatively regulated antiviral response against SVCV infection. Interesting, our previous studies revealed that nucleotide polymorphisms of piscine histone H2A affect disease resistance of zebrafish and grass carp against bacterial infection (15, 39). Whether there is a correlation between piscine H2A nucleotide polymorphisms and viral infection needs further investigation.

TBK1-IRF3 axis plays a pivotal role in inducing type I IFN signaling. To date, identified regulators of TBK1-IRF3 signaling axis have been shown to regulate innate immune responses against various types of pathogens (7, 40). Since histones act as DAMPs, and can engage PRRs to trigger immune signaling pathways just like PAMPs, how histones regulate TBK1-IRF3 axis remains an important, but less understood question. A study showed that histone H2B interacted with IFI16 (gamma-interferon-inducible protein 16) and BRCA1 in the nucleus, and viral infections such as EBV, KSHV and HSV-1 induced the cytoplasmic distribution of H2B-IFI16 and H2B-BRCA1 complexes *via* Ran-GTP protein. Importantly, the H2B-IFI16-BRCA1 complex caused TBK1 and IRF3 phosphorylation and nuclear translocation of pIRF3, which resulted in IFN-β production (41). The significant finding in the present study is that several lines of evidence confirm the negative regulation of piscine histone H2A on the TBK1-IRF3 axis. First, histone H2A inhibited the IFN1 and/or IFN3 promoter activities mediated by TBK1 and IRF3. Second, histone H2A interacted with TBK1 and IRF3 to impair the formation of TBK1-IRF3 complex. Third, histone H2A degraded TBK1 and IRF3 *via* autophagosome-lysosome pathway. Fourth, histone H2A inhibited TBK1-mediated antiviral activity and degraded cytoplasmic and nuclear IRF3 protein. Fifth, inhibition of histone H2A nuclear/cytoplasmic trafficking by Leptomycin B inhibited the protein degradations of TBK1 and IRF3. All these findings together demonstrate a novel negative regulatory feedback loop between cytoplasmic histone H2A and TBK1-IRF3 axis that regulates RLR signaling.

**Figure 8:**
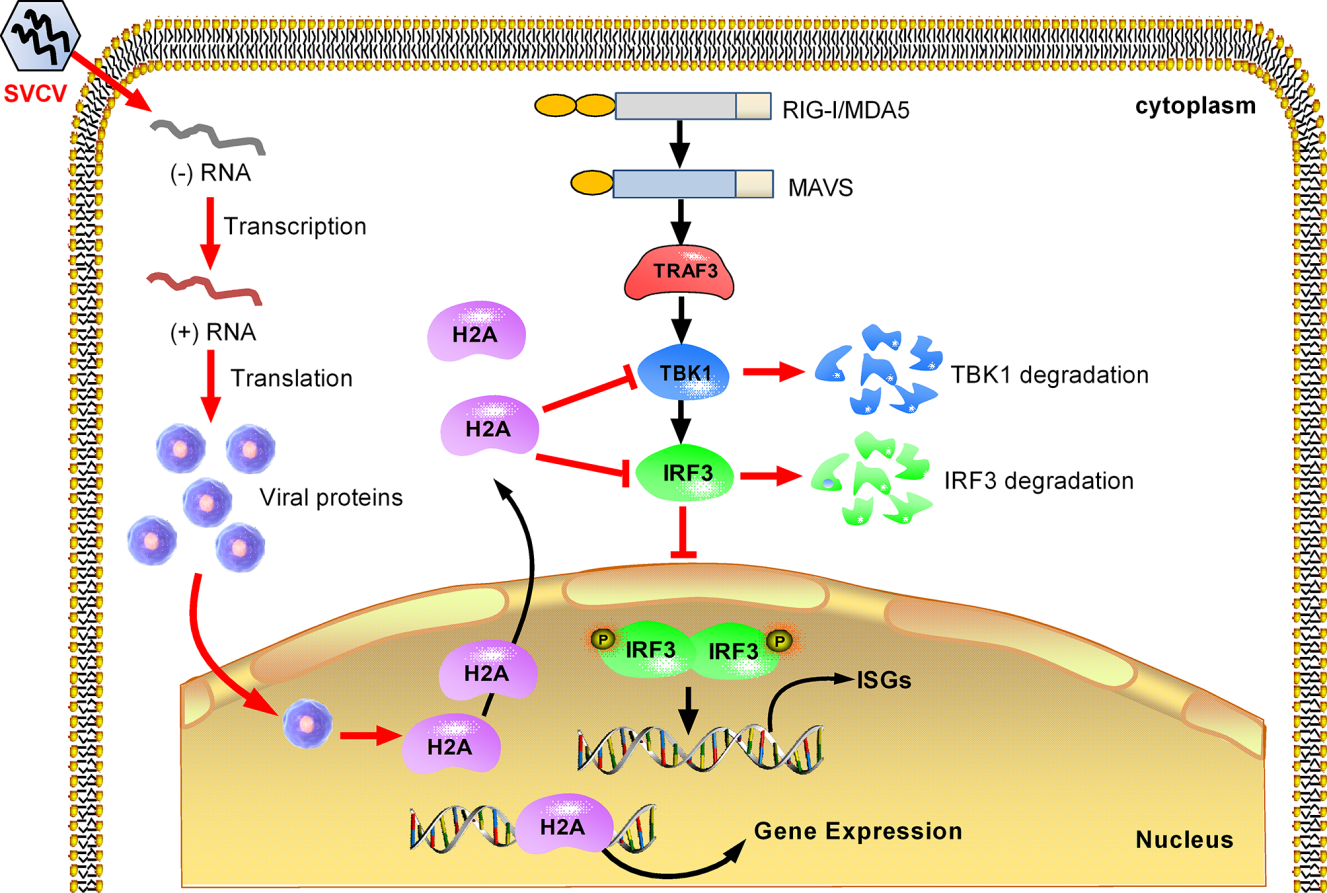
Proposed model illustrating how histone H2A is utilized by SVCV to evade host immune response. Histone H2A is nuclear-localized in the absence of pathogen infection. SVCV infection promotes histone H2A nuclear/cytoplasmic trafficking. In the cytoplasm, histone H2A degrades TBK1 and IRF3, which lead to the impaired formation of TBK1-IRF3 functional complex and the decreased expression of nuclear IRF3 protein. In the cell nucleus, the impaired expressions of IRF3 and/or histone H2A inhibit transcriptional regulation of immune genes, and impede the production of type I IFNs and ISGs in response to SVCV infection.

In summary, we report in this work that piscine histone H2A is critically involved in the negative regulation of TBK1 and IRF3 in antiviral IFN response. Based on the experimental data, a working model is presented for explaining how histone H2A is utilized by SVCV to evade host immune response. Under normal physiological conditions, histone H2A is nuclear-localized. SVCV infection induces the translocation of nuclear histone H2A to the cytoplasm. The cytoplasmic histone H2A targets TBK1 and IRF3 for the protein degradations of TBK1 and IRF3 *via* the lysosomal pathway, which lead to the impaired formation of TBK1-IRF3 functional complex and the decreased expression of nuclear IRF3 protein. Moreover, cytoplasmic histone H2A also blocks TBK1-mediated antiviral activity. All these together impair the antiviral immune response in response to SVCV infection (**Figure 8**). Further work is needed to assess the mechanism of histone H2A nuclear/cytoplasmic trafficking following infection.

## Data Availability Statement

The original contributions presented in the study are included in the article/**Supplementary Material**. Further inquiries can be directed to the corresponding author.

## Ethics Statement

All animal experiments were conducted in accordance with the Guiding Principles for the Care and Use of Laboratory Animals and were approved by the Institute of Hydrobiology, Chinese Academy of Sciences (Approval ID: IHB 2013724).

## Author Contributions

MXC conceived and designed the experiments. XMW, HF and JZ performed the experiments and analyzed the data. MXC, XMW, JZ and YHB interpreted data for the work. MXC and XMW wrote the manuscript. MXC and YHB revised the manuscript. All authors contributed to the article and approved the submitted version.

## Funding

This work was funded by the National Natural Science Foundation of China Grant (31873046), Wuhan Application Foundation Frontier Project (2019020701011467), and Strategic Priority Research Program of the Chinese Academy of Sciences Grant XDA24010308.

## Conflict of Interest

The authors declare that the research was conducted in the absence of any commercial or financial relationships that could be construed as a potential conflict of interest.

## Publisher’s Note

All claims expressed in this article are solely those of the authors and do not necessarily represent those of their affiliated organizations, or those of the publisher, the editors and the reviewers. Any product that may be evaluated in this article, or claim that may be made by its manufacturer, is not guaranteed or endorsed by the publisher.
