# Supplementary figures and images for: Histone H2A Nuclear/Cytoplasmic Trafficking Is Essential for Negative Regulation of Antiviral Immune Response and Lysosomal Degradation of TBK1 and IRF3
Source: Front Immunol. 2021 Nov 18;12:771277. doi: 10.3389/fimmu.2021.771277 (PMC8636446; doi:10.3389/fimmu.2021.771277)

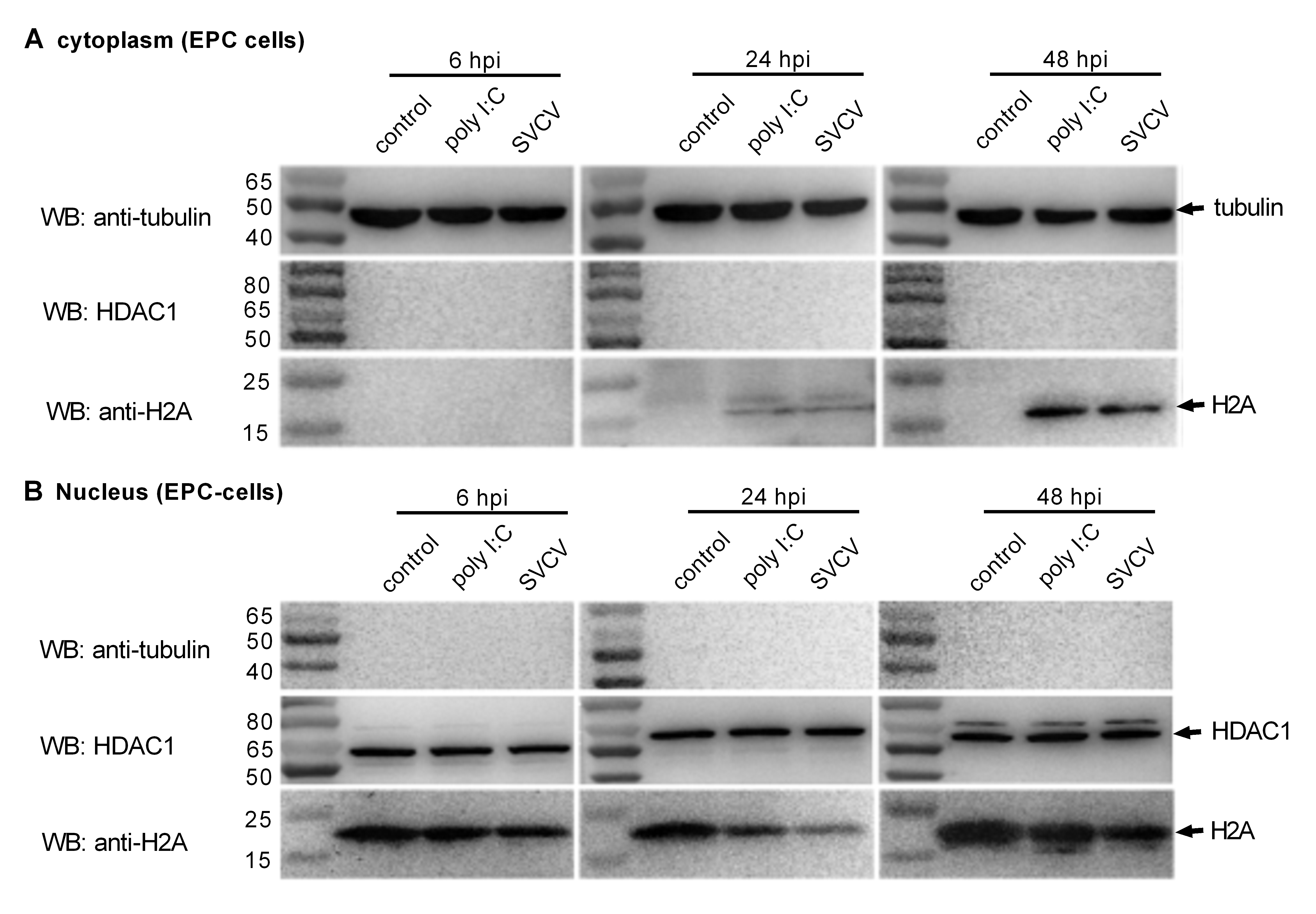

Supplement: Supplementary file 1 [file Image_1.tif]
